# Development of Buffalo Hump in the course of antiretroviral therapy including raltegravir and unboosted atazanavir: a case report and review of the literature

**DOI:** 10.1186/1752-1947-5-70

**Published:** 2011-02-17

**Authors:** Giancarlo Ceccarelli, Gabriella d'Ettorre, Francesco Marchetti, Cecilia Rizza, Claudio M Mastroianni, Bruno Carlesimo, Vincenzo Vullo

**Affiliations:** 1 Department of Infectious Diseases and Public Health, "Sapienza" University, Rome, Italy; 2 Department of Plastic Surgery, "Sapienza" University, Rome, Italy

## Abstract

**Introduction:**

The availability of raltegravir plus atazanavir provides an alternative antiretroviral strategy that may be equally efficacious and less toxic than those currently recommended in HIV treatment guidelines. In fact, this new combination antiretroviral therapy attracts the attention of the scientific community because both drugs have a good safety profile coupled with potent antiviral activity, and their combined use would avert nucleoside- and ritonavir-related toxicities.

**Case presentation:**

We describe the case of a 47-year-old, Caucasian woman treated for HIV-1 infection who developed Buffalo Hump during antiretroviral therapy, including raltegravir and unboosted atazanavir. Clinical evaluation and an ultrasonography scan of the cervical region showed a new progressive increase of lipohypertrophy and the results of DEXA confirmed these data. In our patient the worsening of the Buffalo Hump cannot be attributed to hypercortisolism; insulin-resistance, diabetes, dyslipidemia, hyperlactatemia and metabolic syndrome were not present. Moreover, she was not in therapy with antiretroviral drugs that are described as the cause of Buffalo Hump; on the other hand she developed this side effect three months after the switch of the antiretroviral therapy to raltegravir plus unboosted atazanavir.

**Conclusion:**

Current data indicate that the etiology of HIV-associated Buffalo Hump remains elusive but is likely multifactorial; a possible contributing cause, but not the main cause, could be exposure to antiretroviral drugs. To the best of our knowledge, this is the first report on development of Buffalo Hump in the course of antiretroviral therapy, including the use of these drugs. On the basis of our data we can formulate the hypothesis of a pharmacological pathogenesis that underlies the development of this case of Buffalo Hump in the absence of other risk factors.

## Introduction

Antiretroviral (ARV) treatment guidelines currently recommend ARV regimens containing a Nucleos(t)ide Reverse Transcriptase Inhibitors (N(t)RTIs) based backbone with a Non Nucleoside Reverse Transcriptase Inhibitor (NNRTI) or ritonavir boosted Protease Inhibitor (PI/r). However, significant toxicity has been associated with N(t)RTI(s) and PI/r containing regimens. Recent data presented by Gupta *et al. *show that the combination of raltegravir (RAL) plus unboosted atazanavir (ATV) may be an alternative effective ARV regimen demonstrating good virologic and immunologic response. Furthermore, the combination is well tolerated and has a low incidence of adverse effects [1]. Moreover, side effects reported by Zhu *et al. *during a study in healthy subjects were generally "mild-to moderate" in intensity. Common side effects seen when both drugs were taken were jaundice and headache [2]. Ripamonti *et al. *evidenced that after five to seven months of therapy based on RAL plus ATV no patients discontinued treatment due to drugs used in therapy, adverse events, and no one had a grade 3 or 4 lab toxicity [3]. For these reasons this combination of antiretroviral therapy based on RAL plus ATV attracts the attention of the scientific community because both drugs have a good safety profile coupled with potent antiviral activity, and their combined use would avert nucleoside- and ritonavir-related toxicities.

### Case presentation

We describe the case of a 47-year-old, Caucasian woman treated for HIV-1 infection, who developed buffalo hump during antiretroviral therapy consisting of RAL and unboosted ATV. She was diagnosed with HIV disease in February 1999: the CD4+ cell count was 214/mm3 (11%) and the HIV viral load was 253,200 copies/ml at that stage. An initial highly active antiretroviral therapy (HAART) regimen consisted of zidovudine, lamivudine and indinavir. After 18 months, the therapy was changed to stavudine, lamivudine and nevirapine because of an episode of acute renal colic. Our patient attended outpatient clinics on a regular basis, and was found to have a good immunological and virological response to HAART. By July 2004, she presented with a progressive peripheral fat loss; facial lipoatrophy was apparent but not severe. For these reasons the HAART combination was changed (November 2004), and stavudine was replaced by a nucleotide analogue tenofovir. The CD4+ count was 599/mm3 (16%) and the HIV viral load was < 50 copies/ml at the change of the antiretroviral medications. She was on nevirapine and lamivudine plus tenofovir for five years with a good immune-virological response. We observed neither other fat accumulation nor fat loss and no significant metabolic disorders after the switch. Body Mass Index (BMI), glucose, cholesterol, triglyceride, plasma cortisol and insulin concentrations were normal.

In November 2009 the patient presented with a HIV viral load of 1551 copies/ml; a subsequent test of resistance showed the presence of K65R, K103S, M184V, and G190A. Because she refused therapies with an increased risk of metabolic alterations, the antiviral treatment was changed to RAL 400 mg with unboosted ATV 200 mg twice daily.

**Figure 1:**
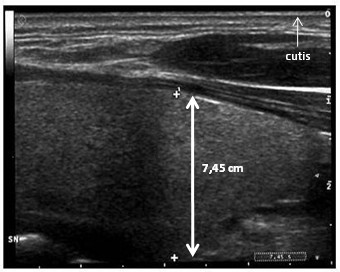
**Ultrasonography scan of the cervical region**. The scan evidenced a massive lipohypertrophy of the dorso-cervical region of the neck (the maximum diameter identified by focusing up and down through the planes of the section was 7.45 cm). The area of fat accumulation was extended over the entire cervical region.

Three months later, it was noted that she developed a new progressive increase of lipohypertrophy of the dorso-cervical region of the neck. There was no localized accumulation of fat in her abdomen and in the submental region of her neck. The hump in the back of her neck was causing neck pain, headaches off and on and sleep apnea. It was causing her discomfort and affecting the motion of her neck. An ultrasonography scan of the cervical region reported a large amount of subcutaneous fat around the posterior aspect of the neck (Figure 1). The results of DEXA confirmed these data. Fasting lipid profile showed a total Cholesterol of 170 mg/dl, HDL-Cholesterol 42 mg/dl and Triglycerides 148 mg/dl. Fasting plasma glucose and response to a glucose tolerance test were normal. Her waist circumference was 80 cm and her BMI was 21. Moreover, there were no significant changes noted in diet, physical activity, and body weight. Thyroid hormones, plasma insulin, cortisol, estradiol, progesterone, prolactin, luteinizing and folliclestimulating hormone concentrations were normal. Her CD4-lymphocyte count was 844/mm3 (20%) and HIV viral load was < 50 copies/ml.

In the next three months she developed a massive lipohypertrophy of the dorso-cervical region of her neck. For this reason the plastic surgery staff proposed surgical removal of the BH due to discomfort, localized pain and the progressive increase of lipohypertrophy of the dorso-cervical region of the neck. The patient refused this option and six months later we observed a stabilization of the subcutaneous fat of the cervical region.

## Discussion

Buffalo Hump is commonly reported in adults with HIV-associated lipodystrophy. Accumulation of fat over the dorso-cervical spine is reported in 2% to 13% of HIV infected patients with a higher prevalence (6 to 13%) in those showing any other feature of the lipodystrophy syndrome [4]. The pathogenesis underlying this aspect of lipodystrophic syndrome is poorly understood. Guallar *et al. *reported that Buffalo Hump adipose tissue shows specific disturbances in gene expression with respect to subcutaneous fat from HIV-1-infected/HAART-treated patients [5]. Some reports indicate that Buffalo Hump is associated with other physical features of the lipodystrophy phenotype and suggest that hyperinsulinemia, insulin resistance, obesity, and hypercortisolism, are important components of this phenotype [6-9]. The close relationship between Buffalo Hump and glycaemic parameters suggests patients with Buffalo Hump are at higher risk for diabetes and metabolic syndrome. In fact, biochemically, patients with Buffalo Hump tend to have or develop signs or symptoms of metabolic syndrome. Mallon *et al. *reported that patients with Buffalo Hump had higher BMI and more total limb and abdominal fat than patients without Buffalo Hump. Current data indicate that a possible contributing cause, but not the main one, could be exposure to antiretroviral drugs: risk factors for Buffalo Hump are longer duration of use of protease inhibitors and longer duration of use of zidovudine [10]. Palacios *et al. *showed that Buffalo Hump was associated with treatment with saquinavir, indinavir, efavirenz, tenofovir and stavudine. Moreover, time of exposure to stavudine and fat loss, one of stavudine's major side-effects, were associated with Buffalo Hump [11]. Previous reports, however, indicated that the appearance of buffalo hump could not be associated with any specific component of HAART regimes and that it is associated with specific disturbances in gene expression of adipose tissue [4,5].

We report the development of the Buffalo Hump cannot be attributed to hypercortisolism; insulin resistance, diabetes, dyslipidemia, hyperlactatemia and metabolic syndrome were not present. Moreover, there were no significant changes noted in our patient's diet, body weight and BMI. Her lifestyle was normal and she followed a regular exercise program.

At the moment she is not in therapy with antiretroviral drugs that are described as the cause of Buffalo Hump; on the other hand she developed this side effect three months after the switch of the antiretroviral therapy to RAL plus unboosted ATV. A caveat of this report is that she had a history of exposure to antiretroviral drugs (Zidovudine, Stavudine, Indinavir) associated with the development of Buffalo Hump. This condition may have predisposed the patient to develop the disorder and could constitute a background that contributes to the final appearance of buffalo hump after raltegravir plus atazanavir treatment. Current data indicate that the etiology of HIV-associated Buffalo Hump remains elusive but is likely multifactorial and includes, metabolic disorders, genetic factors, receipt of ART and HIV infection itself [12].

## Conclusions

The availability of RAL and ATV provides an alternative ARV strategy that may be equally efficacious and less toxic than those currently recommended in HIV treatment guidelines. However, there are few data in the literature available to date regarding such a combination. There are no publications today that describe a relationship between RAL and unboosted ATV therapy and the occurrence of Buffalo Hump. This is the first report on the development of Buffalo Hump in the course of antiretroviral therapy including these drugs: on the basis of our data we can formulate the hypothesis of a pharmacological pathogenesis that underlies the development of this case of Buffalo Hump in absence of other risk factors. More investigation is required to determine if RAL plus unboosted ATV is a safe alternative to RTV boosted PI based ARV strategies and if there are significant side effects related to this ARV treatment.

## Consent

Written informed consent was obtained from the patient for publication of this case report and accompanying images. A copy of the written consent is available for review by the Editor-in-Chief of this journal.

## Competing interests

The authors declare that they have no competing interests.

## Authors' contributions

GC has made substantial contributions to conception and design, acquisition of data, analysis and interpretation of data. GD was involved in drafting the manuscript or revising it critically for important intellectual content. FM and CR made substantial contributions to the acquisition of data. CMM, BC and VV gave final approval of the version to be published. All authors have read and approved the final manuscript.
